# Reliability and validity of the Persian version of psoriatic arthritis screening questionnaire

**DOI:** 10.22088/cjim.14.2.213

**Published:** 2023

**Authors:** Azar Shirzadian Kebria, Abolfazl Eftekhari, Khadijeh Ezoji, Soudabeh Tirgar Tabari, Zeinab Aryanian, Soraya Khafri, Mansour Babaei

**Affiliations:** 1 Department of Dermatology, Clinical Research Development Unit of Ayatollah Rouhani Hospital, Babol University of Medical Sciences, Babol, Iran; 2 Clinical Research Development Unit of Ayatollah Rouhani hospital, Babol University of Medical Sciences, Babol, Iran; 3 Social Determinants of Health Research Center, Health Research Institute, Clinical Research Development Unit of Ayatollah Rouhani Hospital, Babol University of Medical Sciences, Babol, Iran; 4 Autoimmune Bullous Diseases Research Center, Tehran University of Medical Sciences Tehran, Iran; 5 Department of Social Medicine and Health, Babol University of Medical Sciences, Babol, Iran; 6 Mobility Impairment Research Center, Department of Rheumatology, Clinical Research Development Unit of Ayatollah Rouhani Hospital, Babol University of Medical Sciences, Babol, Iran

**Keywords:** Questionnaire, Psoriatic arthritis, Reliability and Validity

## Abstract

**Background:**

The Early Arthritis for Psoriatic Patients (EARP) questionnaire is a fast and simple way to screen psoriatic arthritis. This study was carried out to investigate the diagnostic accuracy of the Persian version of the Early Arthritis for Psoriatic Patients (P-EARP) questionnaire.

**Methods:**

A total of 100 psoriasis patients responded to the questionnaire after the translation procedure (translation, back translation). After determining the validity of the questionnaire, the diagnostic accuracy of the P-EARP questionnaire was assessed using the ROC curve (receiver operating characteristic curve). Internal and external reliability of the questionnaire were also evaluated by statistical tests.

**Results:**

In assessing the reliability of the questionnaire using test-retest, correlation coefficient (r=0.994, p <0.001) and Cronbach's alpha (α = 0.85) were obtained. The P-EARP questionnaire had a sensitivity and specificity of 90.48% and 96.55% in ROC analysis, respectively, and cutoff point 3 was regarded as the cutoff point of the P-EARP questionnaire like the original version of the questionnaire (EARP).

**Conclusion:**

The results of this study showed that the P-EARP questionnaire had high sensitivity and specificity for the identification of psoriatic arthritis. The P-EARP questionnaire is an appropriate screening tool for the identification of psoriatic arthritis in the dermatology clinics.

Psoriatic arthritis is a chronic and potentially debilitating inflammatory arthritis associated with psoriasis that can progress slowly or rapidly and causes joint damage and pain that eventually leads to the patient to disability (1). According to various studies, about 6 to 42% of patients with psoriasis develop psoriatic arthritis and one-third of psoriasis patients with psoriatic arthritis remain undiagnosed for more than two years after experiencing their first symptoms (2). Several factors may delay the diagnosis of psoriatic arthritis, including lack of awareness of the patients of the association between skin and joint symptoms and consequently failure to report past symptoms and signs of the patient, as well as the lack of a specific diagnostic marker (3, 4). Lack of sufficient expertise in the field of musculoskeletal problems among physicians may also be one of the possible reasons for this delay in diagnosis (5). Delay in the diagnosis of psoriatic arthritis may cause joint destruction and disability in a proportion of patients with psoriatic arthritis (6). The EARP questionnaire with high sensitivity and specificity is a simple and easy way to identify arthritis in patients with psoriasis (7). As mentioned above, a suitable tool is needed to be used for psoriatic arthritis screening amongst psoriatic patients. Indeed, no studies have evaluated the properties of this tool among psoriatic patients in Iran. This study aimed to determine the validity and reliability as well as the diagnostic sensitivity and specificity of the Persian version of EARP questionnaire, for the diagnosis of psoriatic arthritis in patients with psoriasis. 

## Methods

This cross-sectional study was performed on 100 psoriasis patients referred to dermatology clinic of Yahyanejad Hospital in Babol during 18 months in 2017-2018. The study population was selected consecutively according to inclusion criteria that patients were diagnosed with psoriasis. Patients with psoriasis coexistent with other inflammatory arthritis, subjects with symptomatic osteoarthritis, septic arthritis, patients with history of inflammatory arthritis or joint surgery, were excluded. After receiving explanations about the purpose of the study, the patients were required to complete informed consent forms. The method was performed in two parts: preparing the Persian version of the questionnaire and then evaluating the diagnostic accuracy of the tool. The EARP Questionnaire consists of 10 questions about the individual’s possible recent episodes of joint swelling, enthesitis, dactylitis, and back and hand morning stiffness occurring during the past 12 months. The questionnaire was composed of dichotomous (yes/no) items; the total score was calculated by summing the score of each question. The proposal of this study was approved by the Ethics Committee of Babol University of Medical Sciences, Babol, Iran, (code: MUBABOL.REC.1396.26)

**Preparation of the Persian version of EARP questionnaire: **At first, the questionnaire was translated into Persian using the standard backward-forward method independently by two Persian translators, a general practitioner and a translator with no background in medical information. These two questionnaires were subsequently transformed into a single questionnaire in a committee consisting of two dermatologists and the two above mentioned translators. Then, two English translators, without any background knowledge in medical information, independently translated the Persian version of the questionnaire into English and a single questionnaire was prepared in English with the exchange of views between the two translators and with the supervision of committee members. Finally, the English version prepared by the researchers and five dermatologists was matched with the original version of the questionnaire and consensus was reached on the existing differences and then it was translated into Persian again. It was tried to use a fluent writing and proper wording in the Persian translation of the EARP questionnaire. The pre-final version of the questionnaire was prepared accordingly. To evaluate the content validity of the experts' opinion about the content coordination of the measurement tool and the purpose of the research, this was used. Experts were asked to provide the necessary feedback after a qualitative review of the tool, based on which corrections were made. Internal consistency was used to assess the internal reliability of the above-mentioned tool. Cronbach's alpha coefficient was calculated and test-retest was used to evaluate the stability of the tool. Then, the pre-final questionnaire was administered to 20 patients and completed by them. The pre-final questionnaire was given to the same patients again 20 days later and was completed by them. Pearson's correlation coefficient between the scores obtained within the 20 days was calculated and paired t-test was used to evaluate the reliability over time, and finally the final version of the questionnaire was prepared.

**Investigating the diagnostic accuracy of the questionnaire:** The Persian version of the EARP (P-EARP) questionnaire was administered to all patients with psoriasis. After filling out the questionnaire, patients were referred to a rheumatologist for clinical confirmation. All patients underwent a complete clinical examination. Radiological and laboratory tests examinations were performed for all patients if clinically indicated. A clinical diagnosis of psoriatic arthritis was confirmed according to CASPAR criteria (The Classification Criteria for Psoriatic Arthritis

**Data Analysis:** The research data were analyzed using the SPSS statistical software, Version 21. The data were analyzed by descriptive statistics, Cronbach’s α, Spearman’s correlation coefficient. Discriminative ability of questionnaire was assessed by application of receiver operating characteristic (ROC) curve analysis. The optimal cutoff value that best distinguished patients with and without psoriatic arthritis was determined at the maximum value of the Yourdon’s index defined as sensitivity + specificity - 1. The overall diagnostic accuracy and predictive ability were estimated based on area under the curve (AUC).

## Results

**Figure 1:**
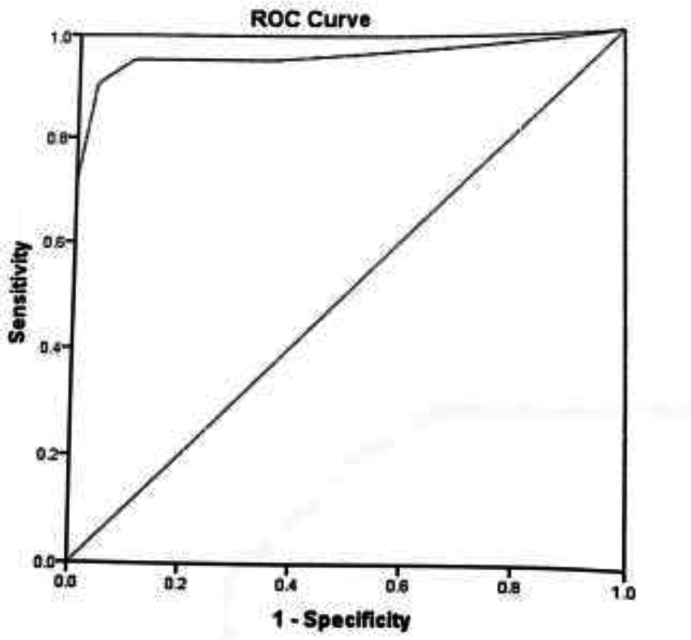
ROC curve of the Persian version of EARP questionnaire

A total of 100 psoriasis patients were studied in the present research as the sample under study. The mean age of the patients was 37.24±12.89 years and 36 (36%) patients were males. The mean age of psoriasis was in the group with psoriatic arthritis 11.49±8.97 and 8.34±8.9 in other patients (P=0.033). 14(50%) patients with arthritis and 14(50%) patients with non-arthritis patients had a family history of psoriasis (P=0.312). From clinical signs, 42(100%) patients with psoriatic arthritis had arthritis symptoms. Dactylitis was observed in 6(14.3%) patients with psoriatic arthritis and was not observed in the other group of patients with psoriasis. Also, 34(81%) patients with psoriatic arthritis had negative RF. Table 1 shows the distribution of samples for some variables. In assessing the internal reliability of the questionnaire using internal consistency, Cronbach's alpha coefficient was calculated as 0.85. In evaluating the stability of the tool using test - retest, Pearson correlation coefficient between the scores obtained within the 20 days of filling the questionnaire by 20 patients (r = 0.994, p <0.001). Paired t-test results showed that there was no significant difference in patients' response to the questionnaire over time (P= 0.083). (Table 2). According to the uni-variant analysis by logistic regression, all questions in the Persian version of the EARP questionnaire (P-EARP) were significantly associated with psoriatic arthritis. (Table 3) Based on the ROC analysis (figure 1) the EARP questionnaire showed an AUC value of 0.961 and at cutoff value of 3 yielded the highest Youden's index value for the diagnosis of psoriatic arthritis at a sensitivity of 90.48% and specificity of 96.55% and accuracy of 93.5%.

**Table 1:** Demographic and background information of the patients under study

		**Psoriasis**	**Psoriatic arthritis**	
Number		58	42	
Sex, n (%)	Woman	34(53.1%)	30(46.9%)	P=0.188
	Man	24(66.7%)	12(33.3%)	
Age , (years;mean±SD)		37.24±12.89	46.74±13.96	P=0.001
Duration of psoriasis , (years;mean±SD)		8.34±8.9	11.49±8.97	P=0.033
Family history of psoriasis, n (%)	Has	14(50%)	14(50%)	P=0.312
	Does not have	44(61.1%)	28(38.9%)	
Arthritis, n (%)		4(6.9%)	42(100%)	_
Nail involvement, n (%)	Has	20(34.5%)	29(69%)	_
	Does not have	38(65.5%)	13(31%)	
Dactylitis, n (%)	Has	0	6(14.3%)	_
	Does not have	58(100%)	36(85.7%)	
Negative rheumatoid factor, n (%)		*-	34(81%)	_
New bone formation around the joint, n (%)		*-	16(34.8%)	_

*These values were not measured for the non-arthritic group.

**Table 2:** Comparison of the results of the Persian version of the EARP questionnaire and the results of the Gold Standard

		**EARP score (n = 100)										
		0	1	2	3	4	5	6	7	8	9	10
*PSA	Has	2	0	2	8	9	6	5	6	3	1	0
	Does not have	37	15	4	2	0	0	0	0	0	0	0

*psoriatic arthritis ** Early Arthritis for Psoriatic Patients

**Table 3:** Multivariate analysis of the Persian version of the Early Arthritis for Psoriatic Patients (P-EARP) questionnaire

**Question**	**Odds ratio (OR)**	**Cl ** **%** **95**	**P-value**
Do your joints hurt?	27.22	9.26-80.06	<0.001
Have you taken analgesics (anti-inflammatory) more thantwice a week for joint pain in the last 3 months?	57	7.21-450.60	<0.001
Do you wake up at night because of low back pain?	4.82	1.68-13.83	0.003
Do you feel stiffness in your hands for more than 30 minutes in the morning?	6.05	1.81-20.26	0.003
Do your wrists and fingers hurt?	23.47	7.67-72.94	<0.001
Do your wrists and fingers swell?	38.76	4.89-307.43	0.001
Does one finger hurt and swell for more than 3 days?	28.5	3.57-227.8	0.002
Does your Achilles tendon (tendon of the instep of the foot) swell?	15.17	1.92-675.82	0.001
Do your feet (from ankle to toe) or ankles hurt?	103.25	15.17-4289.19	<0.001
Does your elbow or hip joint hurt?	28	6.04-129.91	<0.001

## Discussion

The findings of this study indicate that the Persian version of the Early Arthritis for Psoriatic Patients can recognize patients with psoriatic arthritis among patients without arthritis at high sensitivity and specificity. Based on the findings of this study, the diagnostic ability of P-EARP is nearly comparable to CASPAR criteria and thus can be considered as an easy measure for screening psoriatic arthritis. In this study, false negative and false positive results were observed in ~ 9.5% and 3.5% of patients respectively. These findings are in agreement with other studies (4, 7) 

Tinazi et al. (4) also considered the score 3 as the cutoff point in the original version of the tool. Similarly, Maejima et al. considered this cutoff point as the best cutoff point in the Japanese version of the questionnaire as well (7). Compared to original questionnaire (1), the sensitivity of P-EARP is slightly lower (90.48% vs. 91.6%, respectively) but its specificity is substantially higher (96.55% vs. 85.2%, respectively). Similarly, the diagnostic properties are much higher as dicompared to Thi version of questionnaire (1). Maejima et al. produced a Japanese version of this questionnaire in 2015 and provided it to 90 psoriasis patients (7). The Japanese version of EARP had 97.2% and 97.2% sensitivity and specificity, respectively, both of which were higher than the Persian version prepared in this study. Chiowchamwisawakit et al. also prepared a Thai version of the questionnaire in 2016 and provided it to 159 patients. The sensitivity and specificity of 83% and 79.3% were reported respectively for the Thai version of the EARP questionnaire which had less sensitivity and specificity than the Persian version of the questionnaire of this study (1). This difference in the performance of the questionnaires may be due to differences in the characteristics of the study populations including duration of psoriasis, severity of arthritis across various studies (8, 9). The present study had some limitations. The first limitation was the lack of follow-up of patients to be aware of the condition of patients after the diagnosis of psoriatic arthritis. Another limitation was that P-EARP was investigated among psoriatic patients with selected via purposeful sampling. Hence, the findings cannot be generalized to other samples. In conclusion the Persian version of the EARP questionnaire is a reliable and valid tool for the identification of psoriatic arthritis and, given its high sensitivity; it can assist general practitioners and dermatologists in identification of psoriatic arthritis.
